# Changes in Confidence, Feelings, and Perceived Necessity Concerning COVID-19 Booster

**DOI:** 10.3390/vaccines11071244

**Published:** 2023-07-15

**Authors:** Cheryl Lin, Brooke Bier, Ann M. Reed, John J. Paat, Pikuei Tu

**Affiliations:** 1 Policy and Organizational Management Program, Duke University, Durham, NC 27708, USA; c.lin@duke.edu (C.L.); brooke.bier@duke.edu (B.B.); 2 School of Medicine, Duke University, Durham, NC 27708, USA; ann.reed@duke.edu (A.M.R.); john.paat@duke.edu (J.J.P.)

**Keywords:** vaccine hesitancy, trust, risk perceptions, health behavior, public health, communication, Theory of Planned Behavior, Wheel of Emotions, attitude, pandemic

## Abstract

The COVID-19 booster first became available to all adults in the U.S. in November 2021 and a bivalent version in September 2022, but a large population remains booster-hesitant; only 17% of Americans have obtained the updated vaccine as of June 2023. We conducted two cross-sectional surveys in 2021 and 2022 (*n* = 1889 and 1319) to determine whether changes in booster-related feelings or perceptions had occurred and whether they altered vaccination rates over time. We found that both positive and negative emotions had grown stronger between the two years, with the prevalence of annoyance increasing the most (21.5% to 39.7%). The impact of trust on booster intention more than doubled (OR = 7.46 to 16.04). Although perceived risk of infection decreased, more participants in 2022 indicated uncertainty or unwillingness to obtain a new booster than in 2021, while the proportion refusing a booster remained constant at 22.5%. Confidence in the COVID-19 vaccine and feelings of hope from the booster motivated acceptance; both were stronger predictors of booster receptivity than prior vaccination history. Our findings signal a need to rebuild trust by informing people of their continued risk and appealing to positive, especially optimistic emotions to encourage booster uptake. Future research should explore longitudinal trends in behavior and feelings toward new booster doses and the impact of prolonged vaccine hesitancy on infection rates.

## 1. Introduction

In May 2022, the Centers for Disease Control and Prevention (CDC) recommended a COVID-19 booster for all age groups (age five and older), intending to counteract declining vaccine efficacy as new virus strains emerged [1]. Although 80.9% of Americans had obtained at least one original vaccine dose by the year’s end, only about 48% of them received their first booster despite widespread availability [2]. This proportion was even lower for the bivalent booster: fewer than 4% of Americans obtained this updated shot a month after its rollout in September 2022 [3], compared to nearly 9% who received the first-provided vaccine a month post-approval, considering the challenges in supply and distribution noted at the time [4]. Maintaining a high vaccination coverage (plus natural immunity) is key to ending the pandemic and preventing a resurgence [5], but the adoption of the bivalent booster has stalled; the uptake rate reached 15% in the first week of 2023 and had barely passed 17% five months later [2].

Commonly reported concerns with the first booster included fears of safety given its speed of development, feelings that a booster was unnecessary with the protection from the first dosage, or aversion from previous experiences with side effects [6,7,8,9]. These findings reflect those of research on vaccine hesitancy, where risk perception and confidence in a vaccine’s safety or efficacy are primary predictors of acceptance [10,11]. For example, individuals who perceived themselves to be at lower risk of infection were less willing to vaccinate against both influenza and COVID-19 [12,13,14]. Though less is known about the COVID-19 booster, given its relatively recent approval, early studies suggested that booster-related behavior followed a similar pattern, where those without pre-existing conditions or with less concern over becoming ill if they did get COVID were more uncertain about getting boosted [8,15,16].

The reasons for declining or postponing a booster may differ among previously vaccinated individuals [17,18]. People may now perceive less immediate need for the second booster [17], expressing indifference concerning risk or fatigue about the threat of infection as case numbers decrease [2] and the world seemingly returns to its pre-pandemic state [4,19]. Others appear uncertain about the booster itself, distrusting its efficacy, given that new variants are circulating [17] and clinical trials were accelerated [20]. Some individuals are not necessarily rejecting, but rather, are unaware that they are eligible [4], that a second (updated) booster from the same brand of their primary series exists [19], or that it is safe to receive both the flu shot and COVID booster in a short period [17]. Such hesitancies or disinterest starkly contrast with positive reactions toward the prospect of a booster in late 2021, when a majority of vaccinated individuals sought additional protection against the virus’s evolving variants and the perceived severity of the disease [21], indicating plans to get boosted once eligible [6,21,22,23,24,25].

Discrepancies between expressed intent and action are not uncommon in models of preventative health behavior. As described by the Theory of Planned Behavior, choices are often made based on the anticipated outcome-associated emotional effect [26], such that if thinking about a behavior elicits positive emotions, then intentions are likely to predict future behavior. However, intentions as well as related affectual responses are not always stable and may change due to external influences, such as social media or the news [27]. For example, anti-vaccination messaging frequently employs emotional appeals to promote distrust, including underplaying risk of illness or overstating potential side effects [28]. These tactics tend to increase perception of the vaccine’s risk and degrade confidence in it more strongly than infection statistics [29]. Although some scholars explored leveraging positive emotions such as hope, joy, and optimism to motivate pro-vaccination behavior for the first COVID-19 dosage [30,31], none has assessed the roles of these emotions regarding booster behavior.

As the Food and Drug Administration (FDA) plans to roll out booster dosages annually [32], and the recently approved bivalent vaccine [33] may have modified people’s trust of and attitudes toward the booster, understanding the changes in booster perception, as well as behaviors between the first and subsequent dosages, is especially relevant to improve future vaccine acceptance. However, the nuanced emotional experiences elicited by the booster and how those contribute to individuals’ decisions to obtain or reject additional shots remain poorly understood. We conducted two cross-sectional surveys one year apart to compare variations in feelings and confidence between the first and updated boosters. Applying Robert Plutchik’s psychoevolutionary 8-dimentional Wheel of Emotions model [34,35], we examined the presence and effect of different feelings triggered by thinking about the COVID-19 booster. We also investigated the impacts of perceived versus experienced risk associated with uptake intention and action. Our findings could help enhance confidence and urgency to persuade uptake through informed messaging as subsequent COVID-19 boosters are recommended.

## 2. Methods

### 2.1. Participants and Data Collection

We conducted two parallel cross-sectional online surveys in October–November 2021 (shortly before the FDA approved the first booster for all adults) [36] and November–December 2022 (three months after the launch of the bivalent booster) [3] to compare changes in confidence and attitudes concerning the booster. Recruitment and survey distribution were administered through the panel company Centiment (Denver, CO, USA). We used demographic sampling quotas to assemble two nationally representative samples to minimize potential sampling bias. The quotas were based on the 2020 Census data on the composition of the people residing in the U.S.; although the two samples were not an exact match, the large sample size and diversity in demographics relatively resembled the population, indicating the generalizability of the findings. People aged 13 and older on the panel and living in the U.S. were eligible (as per the Children’s Online Privacy Protection Rule [37], adolescents aged 13–17 could participate with parental permission). Individuals were notified of survey availability via email or text and could complete it by phone, tablet, or computer.

Our study protocol was approved by Duke University Institutional Review Board, and consent was obtained from each participant.

### 2.2. Measures

The survey question items were developed through an extensive literature review as well as consultations with major syndicated polls and field experts. Pilot studies were conducted to test the organization and validity of the questions before launching. Slight wording variations were implemented between the two surveys, depending on the respective booster recommendation and availability at the time of the fielding. These differences are noted in the tables presented in the Results section below.

Our primary outcome variables were vaccination status (primary vaccine series for both years and booster uptake in 2022) and booster intention, as assessed by the questions “If and when a COVID-19 booster becomes available to you, would you get it”? in 2021 and, more specifically, “a new booster” in 2022, with answer choices ranging from *very likely* to *not at all likely* (full answer options are listed in the respective tables below).

The key indicators included the following:

- Confidence (“How confident are you with the COVID-19 vaccines (including the updated booster in 2022) available in the U.S.”? from *very* to *not at all confident*).
- Perceived vaccine necessity, according to perceived risk of infection (“How likely do you think you would get COVID-19”? and “If you contracted COVID, how likely is it that you would get very sick”?), experienced risk (“Did you ever get COVID-19”?), and perceived pandemic seriousness (“From what you know, how serious is the current COVID-19 situation”?).
- Prevalent emotions regarding the COVID booster: emotions were first assessed in 2021 by asking participants whether the booster made them feel any of the 24 common emotions (shown in random order) under the eight dimensions described in Plutchik’s Wheel of Emotions [34,38] with a yes/no response. The most prevalent emotion in each of the eight dimensions (annoyance, disapproval, apprehension, sadness, interest, peaceful, surprise, and trust) observed in 2021 were again assessed in our 2022 survey for comparison. Participants in both surveys also were asked how well the words *hope* and *frustrated* described how the booster made them feel (on a scale of 1–10).

Other questions assessed participants’ support of vaccines in general, frequency of obtaining an annual flu shot, whether work or school required COVID-19 vaccination, as well as demographic information.

### 2.3. Data Analysis

We performed chi-square tests to examine differences in vaccination status and booster intention across demographic variables, as well as confidence and perceived necessity between 2021 and 2022. T-tests were performed to compare the means of specified feelings over time. We also calculated odds ratios to estimate the strength of the association between an emotion’s presence and booster uptake. We examined the relationship between primary vaccination behavior and booster intention via Pearson correlations and further analyzed its predictability along with other potential indicators using multiple regression; the variance inflation factor (VIF) was estimated to detect multicollinearity. Analyses were conducted using R Studio 1.3.959 [39].

## 3. Results

Our 2021 survey had 1889 respondents; the 2022 survey had 1319. The distributions in demographic variables roughly represented the U.S. population and were mostly similar between the two surveys, except that the 2022 sample included more females (52.3% vs. 46.5%), Hispanics (15.6% vs. 8.8%), and individuals in the lower income groups; detailed breakdowns by demographics are presented in Table 1.

### 3.1. Vaccination Acceptance and Refusal

The proportion of fully vaccinated (i.e., two doses of Pfizer/Moderna or one dose of Johnson & Johnson) participants increased from 58.1% in 2021 to 65.3% in 2022, aligning with the 7.6% of participants who indicated their intention to get vaccinated in 2021 (Table 2); the share of participants who did not plan to get vaccinated also grew (20.1% to 29.5%). These rates varied by race/ethnicity, with Asian-Americans consistently showing the highest vaccination rates (82.5% in 2021; 87.7% in 2022); the lowest rates occurred among Native Americans in 2021 (40.0%) and whites in 2022 (63.1%). Individuals without a college degree were more likely to reject vaccination in both years (24.0% compared to 12.4% of those with a college degree, *p* < 0.001 in 2021; 34.5% vs 18.6%, *p* < 0.001 in 2022).

### 3.2. Booster Uptake and Intention

A small group of respondents had obtained a booster at the time of the 2021 survey (9.1%), since only high-risk individuals were eligible as of September that year [40]; 49.1% indicated that they would likely get it once eligible. In 2022, 50.4% of the respondents had at least one booster shot (Table 2), with receptivity being higher among the older population: 60.0% of those aged 50 or older were boosted and 52.1% indicated willingness to obtain an updated booster, compared to 48.8% and 45.9% of the younger participants (*p* < 0.001; 0 < 0.01, respectively). Conversely, the proportion of reluctant participants (i.e., “not very likely” to get one) grew from 9.2% to 14.7%; the proportion of participants refusing the booster (“not at all likely”) was identical at 22.5% in both years, with no significant variations between age groups.

Compared by primary series vaccination position, booster intention was the highest in the vaccinated group, although this decreased between the two surveys (from 82.8% to 67.1%); conversely, willingness to obtain a booster by those initially refusing the original vaccine was in single-digit percentage (2.6% to 8.7%). Significantly more participants responded *unsure* or *unlikely to get a booster* in 2022 than 2021 across all vaccination positions (Table 3).

The correlation between self-reported primary vaccine uptake and booster intention remained significant but decreased between 2021 and 2022 (0.728 to 0.617, *p* < 0.001). Booster uptake in 2022 was related to the likelihood of obtaining a future booster (r = 0.637, *p* < 0.001; Table 4).

### 3.3. Perceived and Experienced Risk of COVID-19

**Figure 1:**
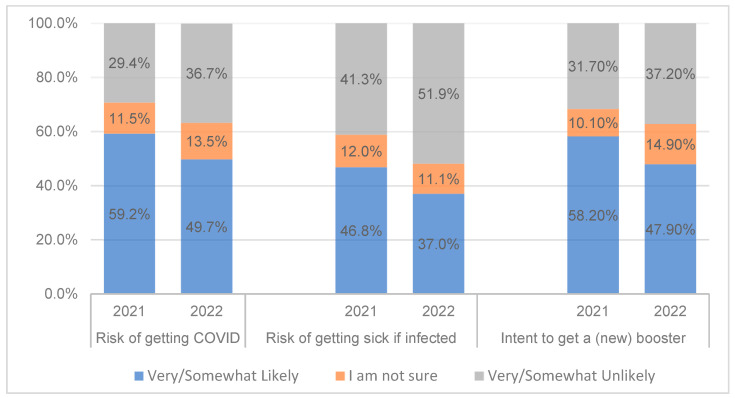
Decreases in perceptions of risk and intention to obtain the booster (n = 1889 in 2021 and n = 1319 in 2022).

In 2022, respondents perceived a lower risk of getting COVID-19 (X^2^ = 5.29, df = 1, *p* < 0.05) and a lower likelihood of getting very sick if they did get infected (X^2^ = 6.04, df = 1, *p* < 0.05) compared to 2021 (Figure 1). Correlations of risk perceptions with primary series vaccination uptake, booster intention, and booster uptake also decreased but stayed significant across the two surveys (Table 4). On the other hand, having become infected with COVID-19 (experienced risk) showed small but statistically significant correlations with primary series uptake in both years and booster intention in 2021 but not with booster uptake and booster intention in 2022, indicating a general decrease in perceived necessity for additional shots. Believing that the pandemic was still a serious situation was more strongly correlated with booster intention in 2022 than 2021, but the share of participants indicating this dropped by nearly half in 2022, from 46.6% to 26.0% (X^2^ = 236.47, df = 1, *p* < 0.001).

### 3.4. Confidence and Hope

Although confidence in the COVID-19 vaccine (including the booster in our second survey) decreased (X^2^ = 101.34, df = 1, *p* < 0.001), the majority of participants were very or somewhat confident in both samples (67.2%; 61.4%; Table 5). Confidence had a consistently strong association with primary vaccination and an even stronger association with booster intention across the two surveys. Confidence was also significantly related to booster uptake in 2022, though at slightly lower strength (Table 4). Further, the widespread discussion about boosters in the media did not significantly change vaccine confidence. However, the booster had brought more hope in 2021 than in 2022 (mean = 6.22 vs 5.65 out of 10, t = 3.283, *p* = 0.001). There was no significant difference in the level of frustration between the two years (Table 5).

### 3.5. Emotions Elicited by the Booster

**Figure 2:**
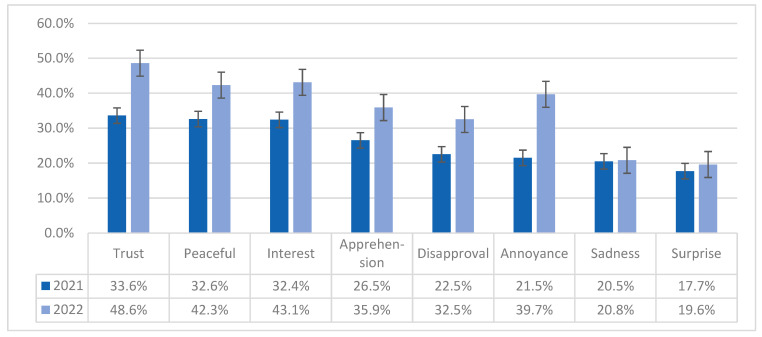
Changes in emotions toward COVID-19 booster (n = 1889 in 2021 and n = 1319 in 2022) *. * Participants were asked “how does the COVID-19 booster make you feel … do you feel (a particular emotion)”? These eight emotions were the most salient in each of the eight dimensions of Plutchik’s Wheel of Emotions, in the order of prevalence observed in 2021.

Six of the eight most frequently felt emotions toward the booster in 2021 grew stronger in 2022, with annoyance increasing the most (184.7%); sadness and surprise remained at the same low levels (Figure 2). Their influence on uptake behavior varied over time. In 2021, participants who expressed trust in the booster were more likely to get boosted (OR = 7.46, 95% CI = 5.85–9.61, *p* < 0.001) than those who did not express trust; this impact more than doubled in 2022 (OR = 16.04, 95% CI = 12.289–21.123, *p* < 0.001). In contrast, in 2021, those that felt disapproval (OR = 0.082, 95% CI = 0.062–0.108, *p* < 0.001), annoyance (OR = 0.235, 95% CI = 0.186–0.298, *p* < 0.001), or apprehension (OR = 0.529, 95% CI = 0.430–0.650, *p* < 0.001) were less likely to obtain the booster. These values increased slightly in 2022, but the differences were not statistically significant.

### 3.6. The Significance of Feeling Hope, Confidence, and Vaccination History

We ran multiple regression models to determine the relative importance of the factors influencing participants’ intention to obtain a (new) booster. After several iterations, the selected model included primary series vaccination status (prior vaccination history), confidence, feelings of hope and frustration, general support of vaccines, work or school vaccination requirement, perceived seriousness of COVID-19, perceived risk of getting COVID-19, perceived likelihood of getting sick if infected, having had COVID, having loved ones who were infected with or died from COVID, and age 65 or older as independent variables. The model yielded an adjusted R^2^ = 0.672, F(df 12, 1230) = 213.166, *p* = 0.000. To be statistically parsimonious, we removed the last four variables that were insignificant. The variance inflation factors (VIFs) of the remaining predictors were between 1.04 and 2.73, indicating low multicollinearity [41].

**Figure 3:**
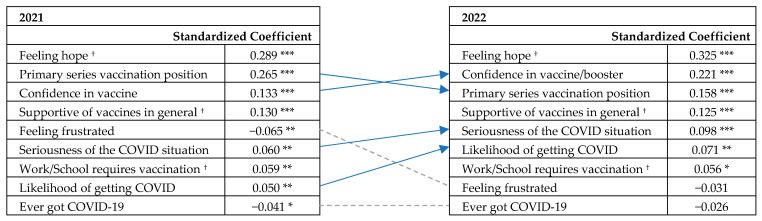
Shifts in the relative importance of indicators of COVID-19 booster intention (by multiple regression). ^†^ The relative importance of variables in predicting booster intention stayed the same from 2021 to 2022. An arrow indicates an increase or decrease in relative importance over time. A dotted line denotes that a factor became insignificant in 2022. Factors are listed in order of importance in the respective years. * Significant at *p* < 0.05; ** significant at *p* < 0.01; *** significant at *p* < 0.001.

The model fit of the revised multiple regression was satisfactory for both 2021 (adjusted R^2^ = 0.672, F(df 9, 1233) = 283.737, *p* = 0.000) and 2022 (adjusted R2 = 0.670, F(df 9, 711) = 163.529, *p* = 0.000). Having felt a sense of hope from the booster was the most important predictor of booster intention in both years, followed by previous COVID vaccination history in the 2021 model and confidence in the booster in the 2022 model (Figure 3). Having ever had COVID-19 had a weak impact in 2021 (β = −0.041, *p* < 0.05) and became insignificant in 2022.

## 4. Discussion

Other than national trackers conducted by major organizations such as the CDC, the Kaiser Family Foundation, and Gallup, we were among the few examining changes in attitudes and psychological responses pertaining to COVID-19 vaccination behavior over time. We observed that booster intention dropped from 2021 to 2022, paralleling decreases in confidence and hopefulness in the booster, along with a perception of less risk concerning the threat and effects of COVID-19. These declines may be attributed to widespread decreases in cases and hospitalizations from 2021 to 2022 [2], as well as environmental signals suggesting a return to a pre-pandemic normalcy (i.e., fewer mask requirements, restrictions on gatherings, etc.) [42]. Our findings may also help explain why bivalent booster uptake has plateaued at around 16% for over four months [2]. The observed pattern, combined with the knowledge that immunity does not last against new strains, is worrisome: the results may suggest a future undesirable trend in vaccination, similar to that associated with the mumps and measles vaccine. For these diseases, low prevalence was previously achieved by successful vaccine programs, but outbreaks reoccurred as low risk perception reduced the perceived need for the vaccine and thus uptake rates [43,44]. These risk assumptions and subsequent behavior underline the critical importance for public health authorities and healthcare professionals to communicate the necessity and distinctive benefits of the booster.

Having ever had COVID was the weakest predictor of booster intention in 2021; this factor became insignificant in 2022. Considering that the majority of Americans have already contracted COVID-19 [2], worries of infection likely diminished due to the mostly mild illness experience, as well as the belief that previous infection provided natural immunity [45,46]. Thus, still un-boosted individuals were not all booster-rejecting; some may have just perceived a low urgency to obtain one. In 2022, more people, both vaccinated and unvaccinated, indicated that they were unlikely or unsure of whether to get the new booster than in 2021; furthermore, a small group in both surveys were unaware of or did not understand the booster. Future vaccination campaigns should aim not only to be educational, but also to provide access and information to individuals that are not cognizant of the potential severity of the disease or their eligibility for/the availability of the vaccine.

In particular, we highlighted the role and strength of emotions in driving health-related behavior. Emotions, both positive and negative, became stronger in 2022 compared to a year ago, likely due to the relatively novel concept of the booster. We illustrated that feelings of hope and confidence in the booster were among the most important indicators of vaccination intention; our second survey showed that they were even more influential than primary vaccine status. These results contrast existing literature which consistently indicated past vaccine behavior to be the most prominent predictor of vaccination intention or uptake [8,47]. Moreover, concerns unique to the COVID-19 booster, such as distrust of accelerated testing [17] or disappointment that a booster does not prevent infection [18], may have strengthened the public’s emotional response and subsequently eroded optimism. These sentiments were echoed in our survey responses, where participants expressed heightened annoyance and persistent frustration from 2021 to 2022, following the release of a new, bivalent booster and initial discussions of a possible annual booster.

Despite the lack of research on booster-related emotions, there is a consensus in the literature that confidence and trust are essential to vaccine acceptance [48,49]. Our data provided further evidence that trust could raise booster acceptance more than seven-fold, with the odds even doubling in 2022. However, building or restoring trust or confidence could be difficult to achieve and is unlikely to be accomplished quickly. We observed an apparent discrepancy between intent and behavior, with confidence showing a stronger association with intention than uptake. Thus, it may be prudent to appeal to positive booster-related feelings, prompting a sense of hope in obtaining a new shot, especially during a crisis, when anxiety about the unknown is prevalent.

We also quantified how disapproval, apprehension, and annoyance predicted lower odds of becoming boosted. Our findings confirmed that the association of negative emotions with a behavior made that behavior less likely in the future [26]. This trend remained stable over the two-year study period, as did the share of participants who expressed no desire to be boosted, emphasizing the influence of emotion on both intent and action. These results also help explain why vaccination history only partially predicted future behavior; we found that the proportion of vaccinated individuals not favoring or unsure of the booster increased more than three times in a year. The group of consistent booster-rejectors also presented a similarity between vaccine and booster decisions: this part of the public has remained resolute in their choice to reject vaccination. Messaging tailored to individuals who planned to be boosted once eligible or were uncertain would likely be most effective, as our results suggest that these groups may be more receptive to such an influence than those who indicated a definite rejection.

### Limitations and Future Research

Our study has various limitations. First, our cross-sectional approach prevented within-subject analysis of perception change, as well as differences between intention and action over time. Future longitudinal studies could more precisely compare booster decisions. Additionally, while we timed the first survey to be before the FDA approval of the first booster for all adults to gauge intention, a small population were already boosted, either because of early eligibility or because they preemptively obtained a third shot. (We grouped these individuals into the “very likely” category in our analysis.) Our second survey was conducted three months after the bivalent booster launch, with time for interested individuals to obtain one; still, a small number might not have been eligible yet because of recent vaccination or infection. Moreover, measuring emotions is complicated. Although we adopted a well-established psychology framework when designing the questionnaire, some of the wording in the survey might not constitute the familiar terms that participants use to describe their feelings, and thus, might not have captured their sentiments. Finally, our use of a self-reported online survey to obtain data inherently introduces biases (e.g., favoring those with access to internet, recall bias), even though it is a practical approach to understand perceptions and behavior with a large sample.

Future research conducted to assess changes or continuities in feelings and attitudes with the development of additional booster shots over multiple years would be valuable. For example, with the upcoming announcement for an annual or second Omicron-specific booster [32,50], it will be critical to explore differences in attitudes and emotions toward that booster and monitor uptake. Comparing booster acceptance to the flu shot and preventing similar fatigue due to the need for repeated vaccinations would also be useful. In addition, the literature would benefit from qualitative inquiries to better understand the emotional experiences we have depicted here. For example, the emotion ‘apprehension’ could mean *doubtful* to one individual, but *curious* to another, warranting future work to explore these nuanced individual differences. Lastly, public health officials should investigate how including emotional appeals (e.g., preventing regret and “boosting” hope alongside trust) in future booster campaigns may drive uptake. As we demonstrated, it is not only important to appeal to positive sentiments, but possibly more so to combat negative feelings and to reduce inaccuracies in perceived risk and necessity.

## Figures and Tables

**Table 1:** Participant characteristics, from two cross-sectional surveys.

Demographics		Survey 1 (2021) n = 1889	Survey 2 (2022) n = 1319
Age	13–15	179 (9.5%)	125 (9.5%)
	16–17	262 (13.9%)	149 (11.3%)
	18–29	343 (18.2%)	252 (19.1%)
	30–39	288 (15.2%)	174 (13.2%)
	40–49	414 (21.9%)	199 (15.1%)
	50–64	272 (14.4%)	228 (17.3%)
	65 and above	131 (6.9%)	192 (14.6%)
Gender most closely identifies with	Female	966 (46.5%)	691 (52.3%)
	Male	879 (51.1%)	610 (46.2%)
	Other/Prefer not to answer	44 (2.3%)	18 (1.4%)
Race/ethnicity best describes you	American Indian/Native American	30 (1.6%)	27 (2.1%)
	Asian/Pacific Islander	110 (5.8%)	57 (4.3%)
	Black/African American	235 (12.4%)	185 (14.0%)
	Hispanic/Latino	167 (8.8%)	209 (15.6%)
	White	1280 (67.8%)	803 (60.9%)
	Other/Prefer not to answer	67 (3.5%)	38 (2.9%)
Educational level	Some high school or less	399 (21.2%)	288 (21.8%)
	High school or equivalent	465 (24.6%)	380 (28.8%)
	Some college or trade school	378 (20.0%)	270 (20.5%)
	College degree	416 (22.0%)	259 (19.6%)
	Graduate/professional degree	225 (11.9%)	100 (7.6%)
	Other/Prefer not to answer	6 (0.3%)	22 (1.7%)
Annual household income (US$) *	<$25,000	251 (21.2%)	336 (32.2%)
	$25,000–$49,999	305 (25.7%)	305 (29.1%)
	$50,000–$99,999	338 (28.5%)	247 (23.6%)
	$100,000–$199,999	209 (17.6%)	99 (9.5%)
	>$200,000	44 (3.7%)	20 (1.9%)
	Prefer not to answer	39 (3.3%)	38 (3.6%)

Note: some categories may not add up to 100% due to rounding. * Participants aged 22 and under were not asked about household income.

**Table 2:** Participant vaccination status and booster intention, from two cross-sectional surveys.

	Survey 1 (2021) n = 1889	Survey 2 (2022) n = 1319
Primary series vaccination status		
Fully vaccinated (2 doses of Pfizer/Moderna or 1 dose of J&J)	1099 (58.1%)	861 (65.3%)
Partially vaccinated (1 dose of Pfizer/Moderna)	84 (4.5%)	47 (3.6%)
Planned to get vaccinated	144 (7.6%)	--
Did not plan to get it ^†^/not vaccinated ^‡^	380 (20.1%)	389 (29.5%)
Not sure ^†^ (still undecided)	182 (9.6%)	--
Unsure ^‡^ (of my vaccination status)	--	22 (1.7%)
Booster status ^‡^		
Non-boosted: did not plan to get boosted		467 (35.4%)
planned to get boosted		137 (10.4%)
Boosted: received 1 booster		255 (19.3%)
received 2 boosters		251 (19.0%)
received 3 or more boosters		159 (12.1%)
Was not sure (of the status)		50 (3.8%)
Booster intention		
“If and when a (new ^‡^) COVID-19 booster becomes available to you, would you get it”?		
I already got a booster shot ^†^	172 (9.1%)	--
Very likely	673 (35.6%)	405 (30.7%)
Somewhat likely	255 (13.5%)	227 (17.2%)
Not very likely	174 (9.2%)	194 (14.7%)
Not at all likely	425 (22.5%)	297 (22.5%)
I am not sure	190 (10.1%)	196 (14.9%)

Note: Some categories may not add up to 100% due to rounding. ^†^ Question wording variation in or addition to the 2021 survey. ^‡^ Question wording variation in or addition to the 2022 survey.

**Table 3:** Changes in intention to get a COVID-19 booster across primary series vaccination status over two years.

Primary Series Position	Vaccinated		Uncommitted *		Did Not Plan to Get Vaccinated	
	2021 n = 1149 (60.8%)	2022 n = 861 (65.3%)	2021 n = 360 (19.1%)	2022 n = 69 (5.2%)	2021 n = 380 (20.1%)	2022 n = 389 (29.5%)
Booster intention ^†^						
Very/somewhat likely to get a booster	82.8%	67.1%	38.6%	29.0%	2.6%	8.7%
Unlikely to get a booster/not sure	7.2%	25.1%	24.2%	43.5%	5.3%	37.0%
Not getting a booster	10.0%	7.8%	37.2%	27.5%	92.1%	54.2%

* Uncommitted: planning to (but not yet) or unsure whether to obtain the vaccine. **^†^** Participants were asked “If and when a (new, in 2022) COVID booster becomes available to you, would you get it”?.

**Table 4:** Factors correlated with COVID-19 vaccination, booster intention, and booster uptake over two years *.

	Primary Series Uptake		Booster Intention		Booster Uptake
	2021	2022	2021	2022	2022
Primary series vaccination uptake	1	1	0.728 ***	0.617 ***	0.610 ***
Booster Intention	0.728 ***	0.617 ***	1	1	0.637 ***
Confidence in COVID-19 vaccine (and booster ^‡^)	0.670 ***	0.650 ***	0.712 ***	0.710 ***	0.562 ***
Emotions toward the booster					
Feeling hope	0.619 ***	0.550 ***	0.731 ***	0.696 ***	0.548 ***
Feeling frustrated	−0.474 ***	−0.237 ***	−0.538 ***	−0.325 ***	−0.217 ***
Supportive of vaccine in general	0.615 ***	0.582 ***	0.655 ***	0.631 ***	0.473 ***
Usually get the annual flu shot	0.401 ***	0.417 ***	0.441 ***	0.506 ***	0.402 ***
Work or school requires COVID-19 vaccination	0.325 ***	0.463 ***	0.330 ***	0.446 ***	0.428 ***
Perceived necessity and risk					
Likelihood of getting COVID-19 without vaccine ^†^ / booster ^‡^	0.448 ***	0.409 ***	0.456 ***	0.391 ***	0.301 ***
Likelihood of getting very sick if infected	0.193 ***	0.084 **	0.260 ***	0.183 ***	0.100 ***
Seriousness of the pandemic	0.332 ***	0.256 ***	0.414 ***	0.428 ***	0.306 ***
Ever got COVID-19	−0.081 **	−0.003	−0.091 ***	−0.041	−0.013
Education level	0.221 ***	0.163 ***	0.174 ***	0.165 ***	0.228 ***
Household income	0.232 ***	0.148 ***	0.171 ***	0.129 ***	0.233 ***

* Pearson correlation significant at *p* < 0.05; ** *p* < 0.01; *** *p* < 0.001 (2-tailed). ^†^ Question wording variation or addition in the 2021 survey, with 1889 participants. ^‡^ Question wording variation or addition in the 2022 survey, with 1319 participants. Note: A pair of bold numbers denotes an increase in correlation from 2021 to 2022 (all other pairs showed a decrease in correlation over time).

**Table 5:** Changes in COVID-19 vaccine confidence, feelings, and assessment of risk.

	Survey 1 (2021) n = 1889	Survey 2 (2022) n = 1319
Vaccine confidence		
“How confident are you with the COVID-19 vaccines (including the booster ^‡^)		
Very confident	737 (39.0%)	446 (33.8%)
Somewhat confident	532 (28.2%)	364 (27.6%)
Not very confident	217 (11.5%)	177 (13.4%)
Not at all confident	312 (16.5%)	237 (18.0%)
I don’t know	91 (4.8%)	95 (7.2%)
“Has the discussion about boosters changed your confidence in the COVID-19 vaccine”?		
A lot more confident	360 (19.1%)	243 (18.4%)
Somewhat more confident	240 (12.7%)	240 (18.2%)
The booster has not changed my opinion about the vaccine	749 (39.4%)	503 (38.1%)
Somewhat less confident	145 (7.7%)	86 (6.5%)
A lot less confident	313 (16.6%)	169 (12.8%)
I have not heard about a booster or don’t understand what it is	82 (4.3%)	78 (5.6%)
Risk assessment		
“From what you know, how serious is the current COVID-19 situation”?		
Very serious	881 (46.6%)	343 (26.0%)
Somewhat serious	648 (34.3%)	396 (30.0%)
A minor concern	269 (14.2%)	365 (27.7%)
Not really a concern	91 (4.8%)	215 (16.3%)
Feelings toward the booster		
“How well do each of these words describe how the COVID-19 booster makes you feel”? (1 = not at all, 10 = extremely)		
	**Mean [SD]**	**Mean [SD]**
Hopeful **	6.22 [3.20]	5.65 [3.33]
Frustrated	4.81 [3.36]	4.47 [3.21]

Note: Some categories may not add up to 100% due to rounding. ** The levels across two years are significantly different at *p* < 0.01. ^‡^ Question wording variation in or addition to the 2022 survey.

## Data Availability

The deidentified data related to this paper is available from the corresponding author one year from the date of publication upon reasonable request with methodologically sound research proposal.
